# The Chance to Become an Elite Athlete After Pediatric And Adolescent Anterior Cruciate Ligament Reconstruction

**DOI:** 10.1177/03635465251320415

**Published:** 2025-03-12

**Authors:** Baldur Thorolfsson, Philipp W. Winkler, Ramana Piussi, Thorkell Snaebjörnsson, Rebecca Hamrin Senorski, Jon Karlsson, Kristian Samuelsson, Eric Hamrin Senorski

**Affiliations:** * Sahlgrenska Sports Medicine Center, Sahlgrenska Academy, University of Gothenburg, Gothenburg, Sweden; † Department of Orthopaedics, Institute of Clinical Sciences, Sahlgrenska Academy, University of Gothenburg, Gothenburg, Sweden; ‡ Department of Orthopaedics and Traumatology, Kepler University Hospital, Johannes Kepler University Linz, Linz, Austria; § Department of Health and Rehabilitation, Institute of Neuroscience and Physiology, Sahlgrenska Academy, University of Gothenburg, Gothenburg, Sweden; ‖ Department of Orthopaedics, Sahlgrenska University Hospital, Sahlgrenska Academy, University of Gothenburg, Mölndal, Sweden; Investigation performed at the Department of Orthopaedics, Institute of Clinical Sciences, Sahlgrenska Academy, University of Gothenburg, Gothenburg, Sweden

**Keywords:** athlete, children, soccer, football, knee injury, return to sport

## Abstract

**Background:**

An anterior cruciate ligament (ACL) injury is a severe condition that may affect the career of young athletes. There is limited evidence on the rate and level of return to sport (RTS) after pediatric and adolescent ACL reconstruction.

**Purpose:**

To evaluate clinical outcomes, the level and rate of RTS, and predictive factors for RTS after pediatric and adolescent ACL reconstruction.

**Study Design:**

Cohort study; Level of evidence, 3.

**Methods:**

Patients aged between 10 and 18 years at the time of primary ACL reconstruction were screened for eligibility. Based on age at the time of ACL reconstruction, patients were divided into the pediatric (female: 11-13 years; male: 11-15 years) and adolescent (female: 14-18 years; male: 16-18 years) groups. Patient-specific, injury-related, and treatment-specific data, as well as subscores of the Knee injury and Osteoarthritis Outcome Score (KOOS) at baseline and 1-, 2-, 5-, and 10-year follow-up, were obtained. A survey consisting of 3 patient-specific and 30 knee-related questions was developed by experts in the management of ACL injuries and was sent to all patients to determine sport-specific variables and RTS rates.

**Results:**

Overall, 1392 patients (total response rate: 24%) were included in this study. There were 81 pediatric patients (mean age at ACL reconstruction, 13.7 ± 1.4 years) and 1311 adolescent patients (mean age at ACL reconstruction, 16.5 ± 1.2 years). Significant improvements in KOOS subscores were observed after both pediatric and adolescent ACL reconstruction at each follow-up time point. After ACL reconstruction, 74% of pediatric patients and 68% of adolescent patients returned to their previous type of sport (*P* = .23). Moreover, 31% of pediatric patients and 23% of adolescent patients became elite athletes (highest national level of junior sport or higher) after ACL reconstruction (*P* = .13). A cartilage injury at the time of ACL reconstruction was found to lower the odds of pediatric and adolescent patients returning to their previous type of sport (odds ratio, 0.60; *P* = .001). A second ACL injury occurred in 25% and 31% of pediatric and adolescent patients, respectively (*P* = .29).

**Conclusion:**

Long-lasting clinical improvements and high RTS rates can be expected after pediatric and adolescent ACL reconstruction. Moreover, young athletes still have the chance to compete at an elite level of sport after ACL reconstruction.

The management of anterior cruciate ligament (ACL) injuries in young patients is challenging, given their anatomy, technical considerations for surgery, and their social context with limited high-quality evidence.^2^ Interestingly, a recent systematic review indicated that ACL reconstruction in skeletally immature patients is a safe and effective procedure that enables a rapid recovery and a return-to-sport (RTS) rate of up to 89%.^14^ Despite significant improvements on various patient-reported outcome measures after ACL reconstruction in young patients, long-lasting reduced knee function has been observed, especially with regard to sport activity and knee-related quality of life.^10^ In addition, growth disturbances and revision rates of up to 28% after pediatric and adolescent ACL reconstruction have been reported in several studies.^10,14,20,21,24^ Worse clinical and functional outcomes and an almost 4-fold increased risk of becoming overweight or obese were found in young patients at 3 to 10 years after a knee injury compared with healthy peers.^22^ Such severe long-term consequences of knee injuries in young patients that could affect their mental health, quality of life, physical activity, and possibly their athletic career require serious attention.^12^

ACL reconstruction is considered the treatment of choice for young patients with recurrent symptomatic knee instability despite ongoing rehabilitation, knee-related athletic restrictions, or concomitant injuries requiring surgical treatment.^2^ Despite encouraging clinical outcomes, recovery after ACL reconstruction requires a considerable amount of time as well as physical and mental resilience for young athletes.^8,13^ Although high RTS rates have been observed after pediatric and adolescent ACL reconstruction, concerns exist on whether young athletes can still compete in elite-level sports after ACL reconstruction.^6^ Hence, it is of high clinical relevance to further investigate RTS variables and factors influencing RTS after pediatric and adolescent ACL reconstruction.

The purpose of this study was to evaluate clinical outcomes, the level and rate of RTS, and predictive factors for RTS after pediatric and adolescent ACL reconstruction. It was hypothesized that pediatric and adolescent ACL reconstruction would result in satisfactory clinical outcomes, a high rate of RTS, and the ability to compete at an elite level and that certain variables affect the rate and level of RTS.

## Methods

This prospective observational cohort study was based on data from the Swedish National Knee Ligament Registry (SNKLR) and was approved by the Swedish Ethical Review Authority (No. 2022-00913-01) and the Ethical Review Board of Gothenburg (No. 2018-091-18). This article was prepared in accordance with the STROBE (Strengthening the Reporting of Observational Studies in Epidemiology) checklist.^19^ The SNKLR is a nationwide database that complies with Swedish legislation for data security and was launched in 2005 and covers >90% of all primary ACL reconstruction cases performed in Sweden.^1^ Data on knee ligament injuries are prospectively collected from operating units and hospitals across Sweden and sent to the SNKLR. Patients and surgeons participate in the SNKLR on a voluntary basis.

The SNKLR aims to improve outcomes for patients with knee ligament injuries by providing clinicians with feedback on clinical outcomes, risk factors, and failure rates after knee ligament reconstruction. Data input in the SNKLR consists of a surgeon-related section and a patient-related section. The treating surgeon records patient-specific, injury-related, and treatment-specific data, while patients are asked to complete the Knee injury and Osteoarthritis Outcome Score (KOOS). The KOOS is a self-administered outcome instrument that has been adopted for a number of languages, including Swedish.^16^ It consists of 5 subscales and 42 questions to evaluate outcomes after a knee injury. Each question has 5 possible answers, which are rated from 0 (no problem) to 4 (extreme problem). The scores for each question are added up for every subscale, ranging from 0 (worst) to 100 (best) points.^17^ Data are collected according to a predefined schedule, starting at baseline (ie, time of ACL reconstruction) and then at 1-, 2-, 5-, and 10-year follow-up.

For the purpose of this study, an additional survey was designed in 2017 to assess sport-specific variables (type of sport, level of sport, etc) and RTS rates in pediatric and adolescent patients from the SNKLR who underwent ACL reconstruction between 2005 and 2022. The newly implemented survey consists of 3 patient-specific and 30 knee-related questions. The survey questions were developed by experts in the management of ACL injuries. The experts included orthopaedic surgeons and physical therapists. The topics and order of the questions were determined by an intensive discussion among the experts. The study-specific survey was used to evaluate RTS rates, the level of sport achieved after ACL reconstruction, the length of time that patients were active within each sport, knee function achieved after ACL reconstruction, and whether patients perceived that they could play their sport as well as before the ACL injury. Each of these variables was assessed with a specific question. Key questions from the study-specific survey can be found in Table 1. The entire survey can be found in Appendix 1 (Swedish version) and Appendix 2 (English version) (both available in the online version of this article).

**Table 1:** Key Questions From Study-Specific Survey^*a*^

Question	Answers
What is the absolute highest level that you were competing in BEFORE your ACL injury?	• International competition, national team, world elite
	• National elite (highest in your country)
	• Elite (national classified leagues under the highest league, such as junior elite)
	• Active competition nonelite
	• Motion and recreation
What is the absolute highest level that you have been competing in AFTER your ACL injury?	• International competition, national team, world elite
	• National elite (highest in your country)
	• Elite (national classified leagues under the highest league, such as junior elite)
	• Active competition nonelite
	• Motion and recreation
	• Could never return after the ACL injury
Do you believe that your knee injury and knee surgery have prevented you from playing sport at a higher level?	Yes/no
Have you suffered a new ACL injury on the same knee since your first ACL injury?	Yes/no
Since your first ACL injury, have you suffered a new ACL injury on your other knee?	Yes/no
What was your main sport/discipline before you suffered your first ACL injury?	Selection of >100 disciplines
Have you returned to the sport that you were active in before your first ACL injury?	Yes/no
If you answered “no” to the previous question, what is the main reason for you not to return to the sport that you were active in before your ACL injury?	• Physical limitations due to the knee injury, such as pain, instability, and poor knee function
	• Fear of a new knee injury
	• Studies, work, or family reasons
	• Other (please specify)
If you could return to the sport that you were active in before your ACL injury, could you perform as good or better compared with before your ACL injury?	Yes/no
Do you still participate in the sport that you were active in before your ACL injury?	Yes/no

a ACL, anterior cruciate ligament.

### Study Population

Patients aged between 10 and 18 years at the time of primary ACL reconstruction who were registered in the SNKLR were screened for eligibility. The study-specific survey had to be fully completed for inclusion in the study. Patients with concomitant fractures as well as nerve, blood vessel, or tendon (ie, injury to the patella, quadriceps tendon, or biceps femoris tendon) injuries were excluded from the study. Additional exclusion criteria were the use of allografts, synthetic grafts, or ACL repair; >48 months between the ACL injury and ACL reconstruction; and age <20 years at the time of survey registration.

The included patients were divided into the pediatric and adolescent groups. The pediatric group comprised female patients aged 11-13 years and male patients aged 11-15 years at the time of ACL reconstruction. The adolescent group comprised female patients aged 14-18 years and male patients aged 16-18 years at the time of ACL reconstruction. The separation into different age groups between female and male patients was based on previous studies and aimed to form one group with open physes and one group with closed physes at the time of ACL reconstruction.^4,6,10^

### Data Collection and Outcome Measures

Patient-specific, injury-related, and treatment-specific data of the included patients were extracted from the SNKLR. In addition, subscores from the KOOS subscales of Symptoms, Pain, Activities of Daily Living, Sport and Recreation, and Knee-Related Quality of Life were extracted from the SNKLR at baseline and 1-, 2-, 5-, and 10-year follow-up. Potential factors associated with successful RTS and to become an elite athlete after ACL reconstruction were determined based on the questions of the study-specific survey.

An elite athlete was defined as answer 1 (international competition, national team, world elite), answer 2 (national elite [highest in the country]), or answer 3 (elite [national classified leagues under the highest league, such as junior elite]) to question 4 of the study-specific survey (Table 1 and Appendices 1 and 2): “What is the absolute highest level that you have been competing in after your ACL injury?”

### Statistical Analysis

SAS/STAT statistical software (Version 9.4; SAS Institute) was used to perform statistical analyses. For categorical variables, the proportion (%) and count (n) were presented. For continuous variables, the mean and standard deviation were presented. For comparisons between groups, the Fisher nonparametric permutation test was used for continuous variables, and the Fisher exact test (lowest 1-sided *P* value multiplied by 2) was used for dichotomous variables. The chi-square test was used for nonordered categorical variables, and the Mantel-Haenszel chi-square test was used for ordered categorical variables. The 95% confidence interval (CI) for the mean difference between groups was determined with the Fisher nonparametric permutation test. Logistic regression analysis was used to assess whether age group (pediatric or adolescent) affected the odds of returning to the sport that patients were active in before the ACL injury as well as if age group affected the odds of becoming an elite athlete after ACL reconstruction. Analysis was adjusted for the variables of sex, weight, height, posterior cruciate ligament injury, medial collateral ligament injury, lateral collateral ligament injury, posterolateral corner injury, medial meniscal injury, lateral meniscal injury, cartilage injury, and graft choice as potential prognostic factors. Results for logistic regression analysis were presented as the odds ratio with 95% CI and *P* value. The significance level for all statistical analyses was set at *P* < .05.

## Results

In total, 1026 patients had to be excluded because of concomitant fractures (n = 6); tendon injuries (n = 1); use of allografts, synthetic grafts, or ACL repair (n = 3); >48 months between the ACL injury and ACL reconstruction (n = 21); age <10 years (n = 1) or age >18 years (n = 816) at the time of ACL reconstruction; age <20 years when registering the survey (n = 175); and other reasons (n = 3). There were 1392 patients (73% female) with a mean age of 16.4 ± 1.4 years at the time of ACL reconstruction who were included in this study. The total study cohort was divided into 81 (6%) pediatric patients with a mean age of 13.7 ± 1.4 years at the time of ACL reconstruction and 1311 (94%) adolescent patients with a mean age of 16.5 ± 1.2 years at the time of ACL reconstruction. Table 2 presents differences between groups. There were significantly more female patients in the adolescent group than in the pediatric group (76% vs 24%, respectively; *P* < .0001). The mean time between ACL reconstruction and completion of the study-specific survey was 9.7 ± 4.2 years.

**Table 2:** Patient-Specific, Injury-Related, Treatment-Specific, and Sport-Specific Data^*a*^

	Total (n = 1392)	Pediatric (n = 81)	Adolescent (n = 1311)	*P* Value
Female sex	1019 (73)	19 (24)	1000 (76)	<.0001
Age at ACL reconstruction, y	16.4 ± 1.4 (10.0-18.0)	13.7 ± 1.4 (10.0-15.0)	16.5 ± 1.2 (14.0-18.0)	.0002
Body mass index, kg/m^2^	23.8 ± 3.5 (16.0-54.0)	23.5 ± 3.7 (16.0-38.9)	23.8 ± 3.4 (16.7-54.0)	.43
Concomitant injuries				
Posterior cruciate ligament	6 (0.4)	00 (0.0)	6 (0.5)	>.99
Medial collateral ligament	44 (3.2)	3 (3.7)	41 (3.1)	.96
Lateral collateral ligament	12 (0.9)	00 (0.0)	12 (0.9)	.97
Posterolateral corner	6 (0.4)	00 (0.0)	6 (0.5)	>.99
Medial meniscus	328 (23.6)	19 (23.5)	309 (23.6)	>.99
Lateral meniscus	391 (28.1)	28 (34.6)	363 (27.7)	.23
Cartilage	202 (14.5)	7 (8.6)	195 (14.9)	.15
ACL graft^*b*^				.052
Hamstring tendon autograft	1282 (92.1)	81 (100.0)	1201 (91.6)	
Patellar tendon autograft	63 (4.5)	00 (0.0)	63 (4.7)	
Other autograft	25 (1.8)	00 (0.0)	25 (1.9)	
Highest sport level before ACL injury^*c*^				.18
International competition, national team, world elite	37 (2.7)	00 (0.0)	37 (2.8)	
National elite (highest in country)	76 (5.5)	3 (3.7)	73 (5.6)	
Elite (national classified leagues under highest league, such as junior elite)	421 (30.2)	25 (30.9)	396 (30.2)	
Active competition nonelite	742 (53.3)	44 (54.3)	698 (53.2)	
Motion and recreation	86 (6.2)	6 (7.4)	80 (6.1)	
Sport before ACL injury^*d*^				.52
Soccer	712 (51.1)	43 (53.1)	669 (51.0)	
Handball	187 (13.4)	7 (8.6)	180 (13.7)	
Floor/field hockey	132 (9.5)	6 (7.4)	126 (9.6)	
Basketball	60 (4.3)	3 (3.7)	57 (4.3)	
Other	301 (21.6)	22 (27.2)	279 (21.3)	

a Data are expressed as No. (%) or mean ± SD (range). ACL, anterior cruciate ligament.

b Missing data in 22 patients.

c Survey question number 7: “What is the absolute highest level that you were competing in before your ACL injury?” Missing data in 30 patients.

d Survey question number 27: “What was your main sport/discipline before you suffered your first ACL injury?”

**Figure 1.**
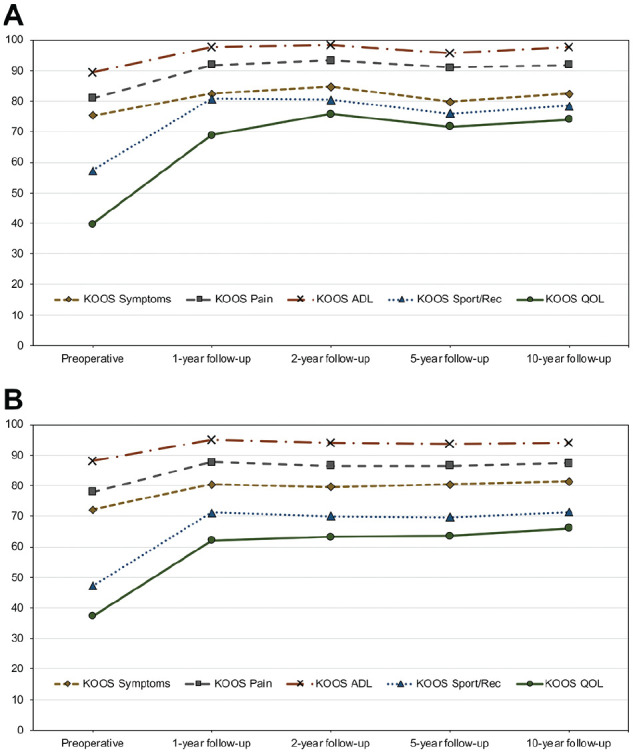
Knee injury and Osteoarthritis Outcome Score (KOOS) subscores before and after (A) pediatric and (B) adolescent anterior cruciate ligament (ACL) reconstruction. ADL, Activities of Daily Living; QOL, Knee-Related Quality of Life; Sport/Rec, Sport and Recreation.

The KOOS subscores were assessed preoperatively and at 1, 2, 5, and 10 years postoperatively for 57, 58, 57, 55, and 31 pediatric patients, respectively, and for 965, 1028, 922, 747, and 418 adolescent patients, respectively. Significant improvements on all KOOS subscales were observed from preoperatively to 1-, 2-, 5-, and 10-year follow-up after adolescent ACL reconstruction (*P* < .0001). After pediatric ACL reconstruction, significant improvements on the KOOS subscales of Activities of Daily Living, Sport and Recreation, and Knee-Related Quality of Life were observed from preoperatively to 1-, 2-, 5-, and 10-year follow-up (*P* < .05). In addition, significant improvements on the KOOS subscale of Symptoms were observed from preoperatively to 2-year follow-up (*P* = .006) as well as on the KOOS subscale of Pain from preoperatively to 1-, 2-, and 5-year follow-up (*P* < .05) after pediatric ACL reconstruction (Figure 1).

**Figure 2.**
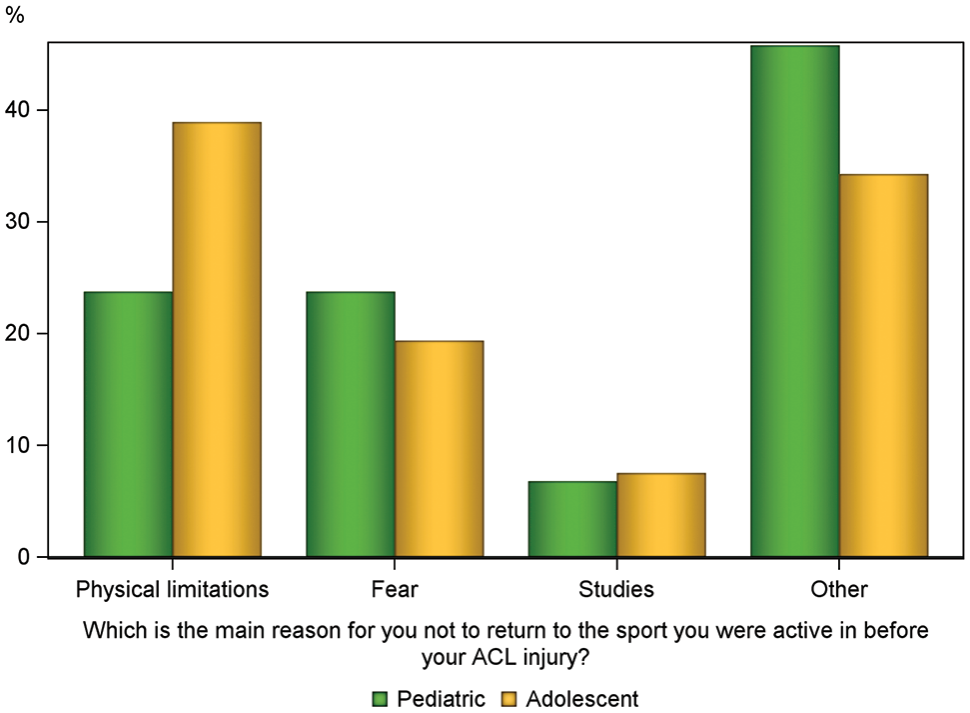
Main reasons for pediatric and adolescent patients not returning to their preinjury sport after anterior cruciate ligament (ACL) reconstruction. Possible answers: physical limitations due to the knee injury, such as pain, instability, and poor knee function; fear of a new knee injury; studies, work, or family reasons; other.

After ACL reconstruction, 74% (n = 60) of the pediatric patients and 68% (n = 886) of the adolescent patients returned to the sport that they were active in before the ACL injury (*P* = .23). At the time of survey completion, 19% (n = 15) of the pediatric patients and 20% (n = 266) of the adolescent patients were still active in their preinjury sport (*P* = .83); 6% (n = 5) of pediatric patients and 8% (n = 105) of adolescent patients were unable to return to sport at all. The main reasons for not returning to the preinjury sport are illustrated in Figure 2. Of note, 54% (n = 44) of pediatric patients and 59% (n = 772) of adolescent patients felt that the ACL injury and subsequent ACL reconstruction prevented them from engaging in sport at a higher level. After ACL reconstruction, 90% (n = 73) of pediatric patients and 86% (n = 1125) of adolescent patients were able to participate in knee-demanding or very knee-demanding activities (*P* = .03).

After ACL reconstruction, 31% (n = 25) of the pediatric patients and 23% (n = 301) of the adolescent patients became an elite athlete (*P* = .13). In addition, 58% (n = 800) of patients reported that they were able to compete at their highest level of sport for <3 years after ACL reconstruction. The time that patients were able to compete at their highest level of sport after ACL reconstruction is shown in detail in Table 3. Pediatric and adolescent patients rated their highest level of knee function after ACL reconstruction to be 87 and 84 of a maximum of 100 points, respectively.

**Table 3:** Time at Highest Sport Level After ACL Reconstruction^*a*^

	Total (n = 1392)	Pediatric (n = 81)	Adolescent (n = 1311)	*P* Value
For how long could you compete at your highest level? (survey question No. 6)				.32
<1 y	253 (18)	16 (20)	237 (18)	
1<3 y	547 (39)	36 (44)	511 (39)	
3<5 y	258 (19)	12 (15)	246 (19)	
≥5 y	334 (24)	17 (21)	317 (24)	

a Data are expressed as No. (%). ACL, anterior cruciate ligament.

Univariable regression analysis demonstrated that a cartilage injury at the time of ACL reconstruction lowered the odds of pediatric and adolescent patients returning to their previous sport (odds ratio, 0.60 [95% CI, 0.44-0.81]; *P* = .001). No factors were found that affected the odds of becoming an elite athlete after pediatric and adolescent ACL reconstruction. Appendix 3 (available online) shows the results of univariable regression analysis in detail.

A second ACL injury was observed in 25% of pediatric patients (20% ACL graft failure, 9% contralateral ACL injury) and 31% of adolescent patients (20% ACL graft failure, 14% contralateral ACL injury) (*P* = .29). The mean time from surgery to the second ACL injury was 36.2 ± 39.2 months for pediatric patients and 37.7 ± 39.6 months for adolescent patients (*P* = .90).

## Discussion

The most important finding of this study was that a large number of pediatric and adolescent patients (74% and 68%, respectively) were able to successfully RTS after ACL reconstruction, and 31% and 23%, respectively, managed to become elite athletes. A cartilage injury at the time of ACL reconstruction was, however, found to lower the odds of RTS after pediatric and adolescent ACL reconstruction. In addition, significant improvements on KOOS subscales lasting up to 10 years after ACL reconstruction were observed in both groups.

In this study, which included 81 pediatric patients and 1311 adolescent patients who underwent ACL reconstruction, there were significant sex differences between groups. In the pediatric group, there were significantly more male than female patients (76% vs 24%, respectively), although significantly more female than male patients were observed in the adolescent group (76% vs 24%, respectively). The observed sex differences are consistent with previous studies, which have reported that ACL injuries in the skeletally immature are more likely to occur in male patients, while female patients are more likely to be injured after physeal closure.^14^ This finding may be related to differences in knee joint laxity, functional performance, psychological factors, and overall risk profiles.^5^

A recent systematic review that evaluated the outcomes of 1691 skeletally immature patients with a mean age of 12.7 years at the time of ACL reconstruction demonstrated significant improvements on patient-reported outcome measures and in knee joint laxity at a mean follow-up of 44.7 months.^14^ The authors of the systematic review indicated that ACL reconstruction in skeletally immature patients is an effective procedure. In the present study, significant improvements on KOOS subscales were observed in both groups after ACL reconstruction at all follow-up time points compared with preoperatively, even at 10 years after ACL reconstruction. This indicates the long-lasting clinical benefit of ACL reconstruction in young patients.

However, a major concern of ACL reconstruction in young patients is the high rate of second ACL injuries. ACL graft failure and contralateral ACL injuries have been reported in 7% to 28% and 8% to 19% of pediatric and adolescent patients, respectively.^9,14,20,21,23^ In the present study, a second ACL injury was observed in 25% of pediatric patients and 31% of adolescent patients, with no significant difference between groups. It is well known that second ACL injuries occur more frequently in pediatric and adolescent patients than in adults.^7,23^ The high rate of second ACL injuries is even more pronounced in young patients who return to high-risk sports.^3,23^ Further risk factors for a second ACL injury in pediatric and adolescent patients include young age and early RTS.^11^ A meta-analysis suggests that young athletes who return to high-risk sports have a 30- to 40-fold increased risk of ACL injuries compared with healthy peers.^23^

Return to physical activity after ACL reconstruction in young patients is a contentious and highly debated topic. Return to any kind of sport has been reported in 89% to 96% of patients after pediatric and adolescent ACL reconstruction.^9,11,14^ However, research has reported that only 45% to 84% of pediatric and adolescent patients return to their preinjury level of sport.^9,11,14^ This is in line with the present study in which 68% to 74% of pediatric and adolescent patients reported that they returned to their preinjury sport after ACL reconstruction. However, >50% of this study’s cohort reported that the ACL injury and subsequent ACL reconstruction prevented them from playing sport at a higher level. It has been reported that young age, early ACL reconstruction, the lack of cartilage injuries, and the patient’s ambition to RTS are predictors of returning to the preinjury level of sport.^15,18^ Consistent with previous studies, the present study found that pediatric and adolescent patients with cartilage injuries at the time of ACL reconstruction were less likely to return to their preinjury sport. Therefore, special attention should be dedicated to the treatment of cartilage injuries in young patients undergoing ACL reconstruction to improve long-term knee health.

Interestingly, one study has reported that up to 81% of pediatric and adolescent patients return to a competitive level of sport.^9^ In the present study, 23% to 31% of patients reported that they managed to become elite athletes after ACL reconstruction. That almost one-third of the patients in this study became elite athletes after ACL reconstruction seems surprisingly high. This may be attributed to the definition of an elite athlete. In this study, competition at an elite level included the 3 highest levels (1: international competition, national team, world elite; 2: national elite [highest in country]; 3: national classified leagues under highest league) in Sweden. It is important to consider that not all of these elite athletes are professional athletes. For example, all athletes of the second highest leagues in soccer and ice hockey are professionals, but most other league athletes in the country are not full professionals. In addition, it is important to note that physical demands at the elite level differ between various sport disciplines. Notably, <10% of this study cohort was not able to RTS at all. Therefore, returning to the desired level of sport or to become an elite athlete after an ACL injury during childhood appears possible. Concomitant soft tissue injuries and modifiable risk factors need to be assessed and, if necessary, addressed to improve clinical outcomes and reduce the risk of second ACL injuries.

### Limitations

Several limitations need to be considered when interpreting the results of this study. First, the data in this study were obtained from the SNKLR and from a study-specific survey sent to 10,200 patients by mail. A total of 2418 patients responded, which gives a total response rate of 24%. The high nonresponse rate increases the risk of selection bias and represents a major limitation of this study. Second, objective data such as isokinetic and isometric strength measurements and time to RTS were not collected, representing important information to evaluate RTS ability in patients after ACL reconstruction. Although a cartilage injury was found to be a negative predictor of RTS, it is unclear how cartilage lesions were treated, which is another limitation of this study. Furthermore, skeletal maturity was assumed based on patient age according to previous studies and not actually assessed by radiography.^4,6,10^ However, the large number of patients in this specific patient population and the long follow-up period underline the strengths of this study. Therefore, the data derived from the large patient cohort included in this study provide new and important information about RTS after ACL reconstruction in pediatric and adolescent patients.

## Conclusion

Long-lasting clinical improvements and high RTS rates can be expected after pediatric and adolescent ACL reconstruction. Moreover, young athletes still have the chance to compete in elite-level sports after ACL reconstruction.

## Supplemental Material

sj-pdf-1-ajs-10.1177_03635465251320415 – Supplemental material for The Chance to Become an Elite Athlete After Pediatric And Adolescent Anterior Cruciate Ligament Reconstruction Supplemental material, sj-pdf-1-ajs-10.1177_03635465251320415 for The Chance to Become an Elite Athlete After Pediatric And Adolescent Anterior Cruciate Ligament Reconstruction by Baldur Thorolfsson, Philipp W. Winkler, Ramana Piussi, Thorkell Snaebjörnsson, Rebecca Hamrin Senorski, Jon Karlsson, Kristian Samuelsson and Eric Hamrin Senorski in The American Journal of Sports Medicine

## References

[1] Ahldén M Samuelsson K Sernert N Forssblad M Karlsson J Kartus J. The Swedish National Anterior Cruciate Ligament Register: a report on baseline variables and outcomes of surgery for almost 18,000 patients. Am J Sports Med. 2012;40(10):2230-2235. 22962296 10.1177/0363546512457348

[2] Ardern CL Ekås G Grindem H , et al. 2018 International Olympic Committee consensus statement on prevention, diagnosis and management of paediatric anterior cruciate ligament (ACL) injuries. Knee Surg Sports Traumatol Arthrosc. 2018;26(4):989-1010. 29455243 10.1007/s00167-018-4865-y PMC5876259

[3] Barber-Westin S Noyes FR. One in 5 athletes sustain reinjury upon return to high-risk sports after ACL reconstruction: a systematic review in 1239 athletes younger than 20 years. Sports Health. 2020;12(6):587-597. 32374646 10.1177/1941738120912846 PMC7785893

[4] Fabricant PD Kocher MS. Anterior cruciate ligament injuries in children and adolescents. Orthop Clin North Am. 2016;47(4):777-788. 27637664 10.1016/j.ocl.2016.05.004

[5] Fältström A Kvist J Bittencourt NFN Mendonça LD Hägglund M . Clinical risk profile for a second anterior cruciate ligament injury in female soccer players after anterior cruciate ligament reconstruction. Am J Sports Med. 2021;49(6):1421-1430. 33856914 10.1177/0363546521999109

[6] Hamrin Senorski E Seil R Svantesson E , et al. “I never made it to the pros…” Return to sport and becoming an elite athlete after pediatric and adolescent anterior cruciate ligament injury: current evidence and future directions. Knee Surg Sports Traumatol Arthrosc. 2018;26(4):1011-1018. 29188332 10.1007/s00167-017-4811-4 PMC5876277

[7] Hopper GP Pioger C Philippe C , et al. Risk factors for anterior cruciate ligament graft failure in professional athletes: an analysis of 342 patients with a mean follow-up of 100 months from the SANTI Study Group. Am J Sports Med. 2022;50(12):3218-3227. 36177758 10.1177/03635465221119186

[8] Ithurburn MP Barenius B Thomas S Paterno MV Schmitt LC. Few young athletes meet newly derived age- and activity-relevant functional recovery targets after ACL reconstruction. Knee Surg Sports Traumatol Arthrosc. 2022;30(10):3268-3276. 34762143 10.1007/s00167-021-06769-4

[9] Kay J Memon M Marx RG Peterson D Simunovic N Ayeni OR. Over 90 % of children and adolescents return to sport after anterior cruciate ligament reconstruction: a systematic review and meta-analysis. Knee Surg Sports Traumatol Arthrosc. 2018;26(4):1019-1036. 29332225 10.1007/s00167-018-4830-9

[10] Kooy C Jakobsen RB Fenstad AM , et al. Major increase in incidence of pediatric ACL reconstructions from 2005 to 2021: a study from the Norwegian Knee Ligament Register. Am J Sports Med. 2023;51(11):2891-2899. 37497771 10.1177/03635465231185742 PMC10478322

[11] Lorange JP Senécal L Moisan P Nault ML. Return to sport after pediatric anterior cruciate ligament reconstruction: a systematic review of the criteria. Am J Sports Med. 2024;52(6):1641-1651. 38299217 10.1177/03635465231187039

[12] Maffulli N Longo UG Gougoulias N Loppini M Denaro V. Long-term health outcomes of youth sports injuries. Br J Sports Med. 2010;44(1):21-25. 19952376 10.1136/bjsm.2009.069526

[13] Mercurio AM Scott EJ Sugimoto D , et al. Assessing the impact of psychological readiness on performance and symmetry in functional testing after ACL reconstruction in pediatric and adolescent patients. Orthop J Sports Med. 2024;12(9):23259671241274768. 10.1177/23259671241274768 PMC11445767 39359482

[14] Migliorini F Cocconi F Schäfer L Memminger MK Giorgino R Maffulli N. Anterior cruciate ligament reconstruction in skeletally immature patients is effective: a systematic review. Knee Surg Sports Traumatol Arthrosc. 2024;32(2):418-431. 38258963 10.1002/ksa.12048

[15] Muller B Yabroudi MA Lynch A , et al. Return to preinjury sports after anterior cruciate ligament reconstruction is predicted by five independent factors. Knee Surg Sports Traumatol Arthrosc. 2022;30(1):84-92. 33885946 10.1007/s00167-021-06558-z

[16] Roos EM Roos HP Ekdahl C Lohmander LS. Knee injury and Osteoarthritis Outcome Score (KOOS): validation of a Swedish version. Scand J Med Sci Sports. 1998;8(6):439-448. 9863983 10.1111/j.1600-0838.1998.tb00465.x

[17] Roos EM Roos HP Lohmander LS Ekdahl C Beynnon BD. Knee injury and Osteoarthritis Outcome Score (KOOS): development of a self-administered outcome measure. J Orthop Sports Phys Ther. 1998;28(2):88-96. 9699158 10.2519/jospt.1998.28.2.88

[18] van Haren I van Cingel REH Verbeek ALM , et al. Predicting readiness for return to sport and performance after anterior cruciate ligament reconstruction rehabilitation. Ann Phys Rehabil Med. 2023;66(3):101689. 35843502 10.1016/j.rehab.2022.101689

[19] von Elm E Altman DG Egger M Pocock SJ Gøtzsche PC Vandenbroucke JP. The Strengthening the Reporting of Observational Studies in Epidemiology (STROBE) statement: guidelines for reporting observational studies. J Clin Epidemiol. 2008;61(4):344-349. 18313558 10.1016/j.jclinepi.2007.11.008

[20] Webster KE Feller JA. Exploring the high reinjury rate in younger patients undergoing anterior cruciate ligament reconstruction. Am J Sports Med. 2016;44(11):2827-2832. 27390346 10.1177/0363546516651845

[21] Webster KE Feller JA Leigh WB Richmond AK. Younger patients are at increased risk for graft rupture and contralateral injury after anterior cruciate ligament reconstruction. Am J Sports Med. 2014;42(3):641-647. 24451111 10.1177/0363546513517540

[22] Whittaker JL Woodhouse LJ Nettel-Aguirre A Emery CA. Outcomes associated with early post-traumatic osteoarthritis and other negative health consequences 3-10 years following knee joint injury in youth sport. Osteoarthritis Cartilage. 2015;23(7):1122-1129. 25725392 10.1016/j.joca.2015.02.021

[23] Wiggins AJ Grandhi RK Schneider DK Stanfield D Webster KE Myer GD. Risk of secondary injury in younger athletes after anterior cruciate ligament reconstruction: a systematic review and meta-analysis. Am J Sports Med. 2016;44(7):1861-1876. 26772611 10.1177/0363546515621554 PMC5501245

[24] Wong SE Feeley BT Pandya NK. Complications after pediatric ACL reconstruction: a meta-analysis. J Pediatr Orthop. 2019;39(8):e566-e571. 10.1097/BPO.0000000000001075 31393290

