# Supplementary material for: The Chance to Become an Elite Athlete After Pediatric And Adolescent Anterior Cruciate Ligament Reconstruction
Source: Am J Sports Med. 2025 Mar 12;53(5):1027–33. doi: 10.1177/03635465251320415 (PMC11951352; doi:10.1177/03635465251320415)
Supplement: sj-pdf-1-ajs-10.1177_03635465251320415 – Supplemental material for The Chance to Become an Elite Athlete After Pediatric And Adolescent Anterior Cruciate Ligament Reconstruction [file sj-pdf-1-ajs-10.1177_03635465251320415.pdf]

The Chance To Become An Elite Athlete After Pediatric And Adolescent ACL Reconstruction

Appendix 1: Study specific survey (Swedish version)

Nr	Fråga	Svarsmöjligheter
1	Förnamn, Efternamn	Fritext
2	Personnummer	12 siffror
3	Kön	Kvinna/man
4	Vilken är den absolut högsta nivån du har tävlat på inom din idrott någonsin EFTER din korsbandsskada?	• Internationell tävlingsnivå/landslag/världselit.<br>• Nationell elit (Högsta serien i landet) (OBS utövar du en sport på nationell nivå, men den sporten du utövar är representativ för internationell världsklass hamnar du i alt.1.)<br>• Elit (Nationellt elitklassificerade ligor under den högsta ligan/tävling junitorelit, ungdomselit)<br>• Aktivt tävlande icke-elit<br>• Motion & rekreation<br>• Kunde aldrig återgå
5	I ditt svar på fråga 4, vilken sport avses?	Val mellan över 100 idrottsgrenar
6	Under hur lång tid spelade/har du nu spelat på din absolut högsta nivå?	• Under 1 år<br>• 1-3 år<br>• 3-5 år<br>• 5 år eller mer
7	Vilken var den absolut högsta nivån du tävlade på inom din idrott FÖRE du fick din ACL skada?	• Internationell tävlingsnivå/landslag/världselit.<br>• Nationell elit (Högsta serien i landet) (OBS utövar du en sport på nationell nivå, men den sporten du utövar är representativ för internationell världsklass hamnar du i alt.1.)<br>• Elit (Nationellt elitklassificerade ligor under den högsta ligan/tävling juniorelit, ungdomselit)<br>• Aktivt tävlande icke-elit<br>• Motion & rekreation
8	På en skala mellan 0% till 100%, om 100% är normalt, var vänlig ange den högsta nivån av knäfunktion du haft sedan in operation.	Fritext
9	Anser du att din knäskada och eventuellt efterföljande operation har hindrat dig från att idrotta på högre nivå?	Ja/Nej
10	Har du ådragit dig en ny främre korsbandsskada på samma knä sedan din första skada?	Ja/Nej
11	Om Ja, när fick du din andra ACL skada? Om du inte vet exakt datum var vänlig ange så noggrant som möjligt.	Fritext

12	Har du opererats för annan knäskada i det knä där du ådrog dig din första korsbandsskada?	Ja/Nej
13	Om Ja på fråga 12, vänligen ange anledning till ny operation, samt sjukhus och om möjligt datum.	Fritext
14	Sedan din första korsbandsskada, har du ådragit dig en främre korsbandsskada på ditt andra knä (motsatt sida där du fick din första korsbandsskada)?	Ja/Nej
15	Om ja på fråga 14, när ådrog du dig denna skada? Datum. Försök ange så exakt som möjligt.	Fritext
16	Om Ja på fråga 14, genomgick du operation för din främre korsbandsskada på det andra knät?	Ja/Nej
17	Have you been treated with surgery for any other injury on the other knee?	Ja/Nej
18	Om Ja på fråga 17, vänligen ange anledning till ny operation, samt sjukhus och om möjligt datum.	Fritext
19	Har någon i din närmaste familj (föräldrar och syskon) ådragit sig en främre korsbandsskada?	Ja/Nej
20	Om ja på fråga 19, vänligen ange vem/vilka av dina familjemedlemmar som också har ådragit sig en främre korsbandsskada?	Inga svarsmöjligheter här
21	Mamma	Ja/Nej
22	Pappa	Ja/Nej
23	En bror	Ja/Nej
24	2 eller fler bröder	Ja/Nej
25	En syster	Ja/Nej
26	2 eller fler systrar	Ja/Nej
27	Vilken var den huvudsakliga idrotten du utövade innan din första främre korsbandsskada?	Val mellan över 100 idrottsgrenar
28	Utövade du den sport du angivit på fråga 27 när du ådrog dig din första korsbandsskada?	Ja/Nej
29	Återgick du till den idrott du angivit på fråga 27 efter din första korsbandsskada?	Ja/Nej
30	Vilken är huvudanledningen till att du inte kunde återgå till den sport du angivit i fråga 27?	• Fysiska begränsningar till följd av din knäskada ex, smärta/svullnad/instabilitet/otillräcklig funktion.<br>• Rädsla för att skada knät igen.<br>• Studier, arbete eller familjeskäl.<br>• Övrigt: ANGE
32	Om du återgick till idrotten du uppgav i fråga 27, upplevde du att du kunde prestera lika bra eller bättre jämfört med innan din främre korsbandsskada?	Ja/Nej
33	Utövar du fortfarande den idrott du angav i fråga 27?	Ja/Nej
34	Vilken är den huvudsakliga anledning till att du inte längre utövar den idrott du angav i fråga 21?	• Fysiska begränsningar till följd av din knäskada ex, smärta/svullnad/instabilitet/otillräcklig funktion.<br>• Rädsla för att skada knät igen.

		• Studier, arbete eller familjeskäl.<br>• Övrigt: ANGE
35	Om du svarade Övrigt på föregående fråga, vänligen utveckla.	Fritext
36	Vilken är den högsta knäkrävande nivå du har utövat idrott/aktivitet på sedan din första främre korsbandsskada? (Frågan avser den nivå du någonsin utsatt ditt knä för sedan din skada, du behöver alltså inte träna på denna nivå för närvarande)?	• Mycket knäkrävande aktiviteter såsom hopp och riktningsförändringar (ex fotboll, basket och handboll.<br>• Knäkrävande aktiviteter såsom ett fysiskt krävande arbete, skidåkning eller tennis.<br>• Något knäkrävande aktiviteter såsom löpning och jogging.<br>• Lätt knäkrävande aktiviteter som promenader, cykling, trädgårdsarbete.
37	På en skala från 1 to 100, är 100 är normalt, skatta din nuvarande knäfunktion.	Fritext
38	Under de senaste 12 månaderna, hur ofta har du utövat följande aktiviteter?	Inga svarsmöjligheter här
39	Löpning	• Mindre än en gang i månaden<br>• En gang i månaden<br>• En gang i veckan<br>• 2-3 gånger i veckan<br>• 4 eller mer gånger i veckan
40	Snabba riktningsförändringar	• Mindre än en gang i månaden<br>• En gang i månaden<br>• En gang i veckan<br>• 2-3 gånger i veckan<br>• 4 eller mer gånger i veckan
41	Löpning med kraftig inbromsning	• Mindre än en gang i månaden<br>• En gang i månaden<br>• En gang i veckan<br>• 2-3 gånger i veckan<br>• 4 eller mer gånger i veckan
42	En snabb vändning (minst 90 grader) med vigten på ditt skadade ben.	• Mindre än en gang i månaden<br>• En gang i månaden<br>• En gang i veckan<br>• 2-3 gånger i veckan<br>• 4 eller mer gånger i veckan
43	Får vi lov att kontakta dig via telefon om vi behöver förtydligande av dina svar? Ange i såfall mobilnummer.	Fritext
44	Tack för att du deltagit! Om du vill ha en summering av dina uppgifter var vänlig ange din mailadress.	Fritext

Appendix 2: Study specific survey (English version)

Nr	Question	Answers
1	First name / last name	Free text
2	Swedish social security number	12 numbers
3	Sex	Male / Female
4	Which is the absolute highest level you have been competing in AFTER your ACL injury?	• International competition; national team; world elite<br>• National elite (highest in your country)<br>• Elite (national classified leagues under the highest league, such as, junior elite)<br>• Active competition non-elite<br>• Motion and recreation<br>• Could never return after ACL injury
5	In relation to the previous question, which sport?	Selection of over 100 disciplines
6	For how long could you compete at your highest level?	• Under 1 year<br>• 1-3 years<br>• 3-5 years<br>• 5 years or more
7	Which is the absolute highest level you have been competing in BEFORE your ACL injury?	• International competition; national team; world elite<br>• National elite (highest in your country)<br>• Elite (national classified leagues under the highest league, such as, junior elite)<br>• Active competition non-elite<br>• Motion and recreation
8	On a scale between 0% to 100%, if 100% is normal, please rate the highest level of knee function you have had since your ACL surgery.	Free text
9	Do you believe that your knee injury and knee surgery has prevented you from sporting at a higher level?	Yes/no
10	Have you suffered a new ACL injury on the same knee since your first ACL injury?	Yes/no
11	If you answered Yes to the previous question, when did you suffer your second ACL injury. Please specify the date if possible (YYYY-MM-DD). If you do not know the exact date please specify month or year.	Free text
12	Have you been treated with surgery for a new knee injury in the same knee as your first ACL injury?	Yes/no
13	If you answered Yes to the previous question, please specify the injury, which hospital and month/year if possible.	Free text
14	Since your first ACL injury, have you suffered a new ACL injury on your other knee?	Yes/no
15	If you answered Yes to the previous question, when did you suffer your ACL injury on the other knee. Please specify the date if possible (YYYY-MM-DD). If you do not know the exact date please specify month or year.	Free text
16	Have you been treated with surgery for your ACL injury on the other knee?	Yes/no
17	Have you been treated with surgery for any other injury on the other knee?	Yes/no

18	If you answered Yes to the previous question, please specify the injury, which hospital and month/year if possible.	Free text
19	Has any of your parents or siblings suffered an ACL injury?	Yes/no
20	If yes to the previous question, please specify which family member/members in the following few questions.	No answer options here
21	Mother	Yes/no
22	Father	Yes/no
23	Brother	Yes/no
24	Two or more brothers	Yes/no
25	Sister	Yes/no
26	Two or more sisters	Yes/no
27	What was your main sport/discipline before you suffered your first ACL injury?	Selection of over 100 disciplines
28	Were you participating in that sport/discipline when you suffered your first ACL injury?	Yes/no
29	Have you returned to the sport you were active in before your first ACL injury?	Yes/no
30	If you answered No to the previous question, which is the main reason for you not to return to the sport you were active in before your ACL injury?	• Physical limitations due to the knee injury such as pain, instability, poor knee function<br>• Fear for a new knee injury<br>• Studies, work or family reasons<br>• Other (please specify in the next question)
31	If you answered Other, in the previous question please specify.	Free text
32	If you could return to the sport you were active in before your ACL injury, could you perform as good or better compared with before your ACL injury?	Yes/no
33	Are you still active in the sport you were active in before your ACL injury?	Yes/no
34	If you answered No to the previous question, which is the main reason for you not to be active in the sport you were active in before your ACL injury?	• Physical limitations due to the knee injury such as pain, instability, poor knee function<br>• Fear for a new knee injury<br>• Studies, work or family reasons<br>• Other (please specify in the next question)
35	If you answered Other, in the previous question please specify.	Free text
36	Which is the highest level of knee-demanding activity you have been active in since your ACL injury? (question refers to the highest level you have ever been active in; you do not need to be active at that level at this moment)	• Very knee demanding activity with pivoting ad jumping (football, basket, handboll)<br>• Knee demanding activity such as hard works, skiing or tennis<br>• Somewhat knee demanding activity such as running or jogging<br>• Light knee demanding activity such as walking, biking or gardening.
37	From a scale between 1 and 100, where 100 is normal, please rate your current knee function.	Free text

38	During the last twelve months, how often have you participated in the following activities?	No answer options
39	Running	• Less than once in a month<br>• Once a month<br>• Once a week<br>• 2-3 times per week<br>• 4 or more times per week
40	Quick cutting or pivoting movements.	• Less than once in a month<br>• Once a month<br>• Once a week<br>• 2-3 times per week<br>• 4 or more times per week
41	Running with quick deceleration or stopping quickly.	• Less than once in a month<br>• Once a month<br>• Once a week<br>• 2-3 times per week<br>• 4 or more times per week
42	Turning quickly (at least 90 degrees) with your body weight on the injured knee.	• Less than once in a month<br>• Once a month<br>• Once a week<br>• 2-3 times per week<br>• 4 or more times per week
43	Thank you for participating. Do you give permission for telephone contact in case any further clarification is wanted about any of your answers? If so, please fill in your telephone number.	Free text
44	Would you like a summary of your answers on e-mail? If so, please fill in your e-mail address.	Free text

Appendix 3:

Univariable regression analysis for survey question 29 “Have you returned to the sport you were active in before your first ACL injury?

Variable	n	n missing	Value	n (%) of event	OR (95%CI)	p-value	Area under ROC-Curve (95%CI)
**Age group**	1392	0	**Pediatric**	60 (74)			
			**Adolescent**	886 (68)	0.73 (0.44-1.22)	0.23	0.51 (0.50-0.52)
**Sex**	1392	0	**Female**	690 (68)			
			**Male**	256 (67)	1.04 (0.81-1.35)	0.75	0.50 (0.48-0.53)
**Weight, [kg]**	1127	265	**36- <65**	259 (67)			
			**65- <75**	261 (71)			
			**75–182**	257 (70)	1.00 (0.99-1.01)	0.55	0.51 (0.47-0.54)
**Height, [cm]**	1121	271	**147- <168**	261 (69)			
			**168- <175**	249 (70)			
			**175–215**	262 (69)	1.00 (0.99-1.02)	0.89	0.51 (0.47-0.54)
**PCL injury**	1392	0	**No**	944 (68)			
			**Yes**	2 (33)	0.23 (0.04-1.28)	0.094	0.50 (0.50-0.51)
**MCL injury**	1392	0	**No**	918 (68)			
			**Yes**	28 (64)	0.82 (0.44-1.53)	0.53	0.50 (0.49-0.51)
**LCL injury**	1392	0	**No**	939 (68)			
			**Yes**	7 (58)	0.66 (0.21-2.08)	0.48	0.50 (0.50-0.51)
**PLC injury**	1392	0	**No**	944 (68)			
			**Yes**	2 (33)	0.23 (0.04-1.28)	0.094	0.50 (0.50-0.51)
**Medial meniscus injury**	1392	0	**No**	733 (69)			
			**Yes**	213 (65)	0.84 (0.64-1.09)	0.18	0.52 (0.49-0.54)
**Lateral meniscus injury**	1392	0	**No**	679 (68)			
			**Yes**	267 (68)	1.02 (0.79-1.31)	0.87	0.50 (0.48-0.53)
**Cartilage injury**	1392	0	**No**	829 (70)			
			**Yes**	117 (58)	0.60 (0.44-0.81)	0.0010	0.53 (0.51-0.55)
**Graft choice**	1370	22	**Hamstring vs Hamstring**	871 (68)	1.00	0.81***	
			**Patella vs Hamstring**	41 (65)	0.88 (0.52-1.50)	0.64	
			**Other vs Hamstring**	18 (72)	1.21 (0.50-2.93)	0.67	0.50 (0.49-0.52)

*All tests are performed with univariable logistic regression. P-values, OR, and Area under ROC-curve are based on original values and not on stratified groups. OR is the ratio for the odds for an increase of the predictor of one unit. ***, p-value for the entire effect/factor/variable. ACL, anterior cruciate ligament; CI, confidence interval; LCL, lateral collateral ligament; MCL, medial collateral ligament; OR, odds ratio; PCL, posterior cruciate ligament; PLC, posterolateral corner.*

Univariable regression analysis to assess whether patients became elite athletes after ACL reconstruction.

	n	n missing	Value	n (%) of event	OR (95%CI)	p-value	Area under ROC-Curve (95%CI)
**Age group**	1370	0	**Pediatric**	25 (31)			
			**Adolescent**	301 (23)	0.68 (0.42-1.11)	0.13	0.51 (0.50-0.53)
**Sex**	1370	0	**Female**	235 (23)			
			**Male**	91 (25)	1.10 (0.84-1.46)	0.48	0.51 (0.48-0.54)
**Weight, [kg]**	1113	257	**36- <65**	92 (24)			
			**65- <75**	84 (23)			
			**75–182**	96 (26)	1.00 (0.99-1.01)	0.51	0.52 (0.48-0.56)
**Height, [cm]**	1107	263	**147- <168**	81 (22)			
			**168- <175**	92 (26)			
			**175–215**	96 (26)	1.01 (0.99-1.02)	0.31	0.52 (0.49-0.56)
**PCL injury**	1370	0	**No**	326 (24)			
			**Yes**	0 (0)	lim(OR)=0	0.39	N/A
**MCL inujury**	1370	0	**No**	313 (24)			
			**Yes**	13 (30)	1.40 (0.72–2.72)	0.32	0.51 (0.49-0.52)
**LCL inujury**	1370	0	**No**	322 (24)			
			**Yes**	4 (33)	1.61 (0.48-5.38)	0.44	0.50 (0.50-0.51)
**PLC inujury**	1370	0	**No**	325 (24)			
			**Yes**	1 (17)	0.64 (0.07-5.49)	0.68	0.50 (0.50-0.50)
**Medial meniscus inujury**	1370	0	**No**	255 (24)			
			**Yes**	71 (22)	0.88 (0.65-1.18)	0.38	0.51 (0.49-0.54)
**Lateral meniscus inujury**	1370	0	**No**	236 (24)			
			**Yes**	90 (24)	1.00 (0.76-1.32)	0.99	0.50 (0.47-0.53)
**Cartilage inujury**	1370	0	**No**	283 (24)			
			**Yes**	43 (22)	0.88 (0.62-1.27)	0.51	0.51 (0.49-0.53)
**Graft choice**	1348	22	**Hamstring vs Hamstring**	294 (23)	1.00	0.28***	
			**Patella vs Hamstring**	19 (31)	1.45 (0.83-2.53)	0.19	
			**Other vs Hamstring**	4 (16)	0.63 (0.21-1.84)	0.40	0.51 (0.50-0.53)

*All tests are performed with univariable logistic regression. P-values, OR, and Area under ROC-curve are based on original values and not on stratified groups. OR is the ratio for the odds for an increase of the predictor of one unit. ***, p-value for the entire effect/factor/variable. ACL, anterior cruciate ligament; CI, confidence interval; LCL, lateral collateral ligament; MCL, medial collateral ligament; OR, odds ratio; PCL, posterior cruciate ligament; PLC, posterolateral corner.*
